# Production of Transgenic Calves Expressing an shRNA Targeting Myostatin

**DOI:** 10.1002/mrd.22007

**Published:** 2011-11-18

**Authors:** K Tessanne, MC Golding, CR Long, MD Peoples, G Hannon, ME Westhusin

**Affiliations:** 1 Department of Veterinary Physiology and Pharmacology, College of Veterinary Medicine, Texas A&M UniversityCollege Station, Texas; 2 Watson School of Biological Sciences, Howard Hughes Medical Institute, Cold Spring Harbor LaboratoryCold Spring Harbor, New York

## Abstract

Myostatin (MSTN) is a well-known negative regulator of muscle growth. Animals that possess mutations within this gene display an enhanced muscling phenotype, a desirable agricultural trait. Increased neonatal morbidity is common, however, resulting from complications arising from the birth of offspring with increased fetal muscle mass. The objective of the current research was to generate an attenuated MSTN-null phenotype in a large-animal model using RNA interference to enhance muscle development without the detrimental consequences of an inactivating mutation. To this end, we identified a series of short interfering RNAs that demonstrated effective suppression of MSTN mRNA and protein levels. To produce transgenic offspring capable of stable MSTN suppression in vivo, a recombinant lentiviral vector expressing a short hairpin RNA (shRNA) targeting MSTN for silencing was introduced into bovine fetal fibroblasts. These cells were used as nucleus donors for somatic cell nuclear transfer (SCNT). Twenty blastocysts were transferred into seven recipient cows resulting in five pregnancies. One transgenic calf developed to term, but died following delivery by Caesarean-section. As an alternative strategy, microinjection of recombinant lentiviral particles into the perivitelline space of in vitro-produced bovine zygotes was utilized to produce 40 transgenic blastocysts that were transferred into 14 recipient cows, resulting in 7 pregnancies. Five transgenic calves were produced, of which three expressed the transgene. This is the first report of transgenic livestock produced by direct injection of a recombinant lentivirus, and expressing transgenes encoding shRNAs targeting an endogenous gene (myostatin) for silencing.

## INTRODUCTION

Myostatin (MSTN), also known as Growth Differentiation Factor 8 (GDF8), is a negative regulator of muscle growth. Breeds of cattle such as the Belgian Blue and Piedmontese, possess mutations within this gene and frequently display dramatically increased muscle mass (Grobet et al., 1997; McPherron and Lee, 1997; Berry et al., 2002). This protein's most notable effect is on the hypertrophy and hyperplasia of muscle fibers, both pre- and post-natally. While a desirable agricultural trait, the positive attributes of this double muscling phenotype are offset by an associated increase in dystocia and decreased fertility (Bellinge et al., 2005). Therefore, there is considerable interest in the potential to modulate the level of myostatin expression in livestock species in a way that increases meat production without the negative attributes associated with natural-occurring myostatin mutations. Animals with reduced, but not eliminated, myostatin should display an increase in muscle mass and potentially avoid the detrimental effects of double muscling, such as decreased fertility and dystocia. Moreover, alteration of livestock myostatin expression could provide new insight into myostatin's role in human muscle cell activation and development (McNally, 2004; Schuelke et al., 2004).

RNA interference (RNAi) provides a means of regulating gene expression by targeting mRNA in a sequence-specific manner and inducing either transcript degradation or translational inhibition. RNAi was first described in detail by Fire and Mello in *C. elegans* in 1998 (Fire et al., 1998). Since then, the RNAi response has been shown to be highly conserved among drosophila (Kennerdell and Carthew, 1998), fungi (Romano and Macino, 1992), plants (Chuang and Meyerowitz, 2000), and mammals (Wianny and Zernicka-Goetz, 2000). Using experimentally delivered short hairpin RNAs (shRNAs) and short interfering RNAs (siRNAs), RNAi has become a powerful genetic tool, not only for understanding basic gene function but also in the development of novel applications for animal agriculture and for animal and human disease.

The development of techniques for producing transgenic livestock that express shRNAs targeting endogenous and/or exogenous genes for silencing has enormous potential for livestock improvement (Long et al., 2010). In addition, introduction of transgenes using viral vectors, in particular lentiviral vectors, has provided an avenue for increasing the efficiency of transgenic livestock production. Combined with assisted reproductive technologies (ARTs) such as somatic cell nuclear transfer (SCNT) and in vitro fertilization (IVF), viral gene transfer has been used successively to produce a variety of transgenic animals, including mice, dogs (Hong et al., 2009), cats (Yin et al., 2008), cows (Hofmann et al., 2004), and pigs (Park et al., 2001, 2002). Here we provide the first report of transgenic calves produced by direct injection of one-cell embryos with recombinant lentivirus expressing an shRNA construct designed to reduce bovine myostatin mRNA and protein expression. Analysis of muscle characteristics in these animals should provide insight into the role of myostatin in muscle generation and development. Moreover, information gained from these experiments could contribute to agriculture applications in addition to the development of human therapeutics for such disorders as muscular dystrophy and sarcopenia.

## RESULTS

### Evaluation of Myostatin mRNA Suppression In Vitro

**Figure 1:**
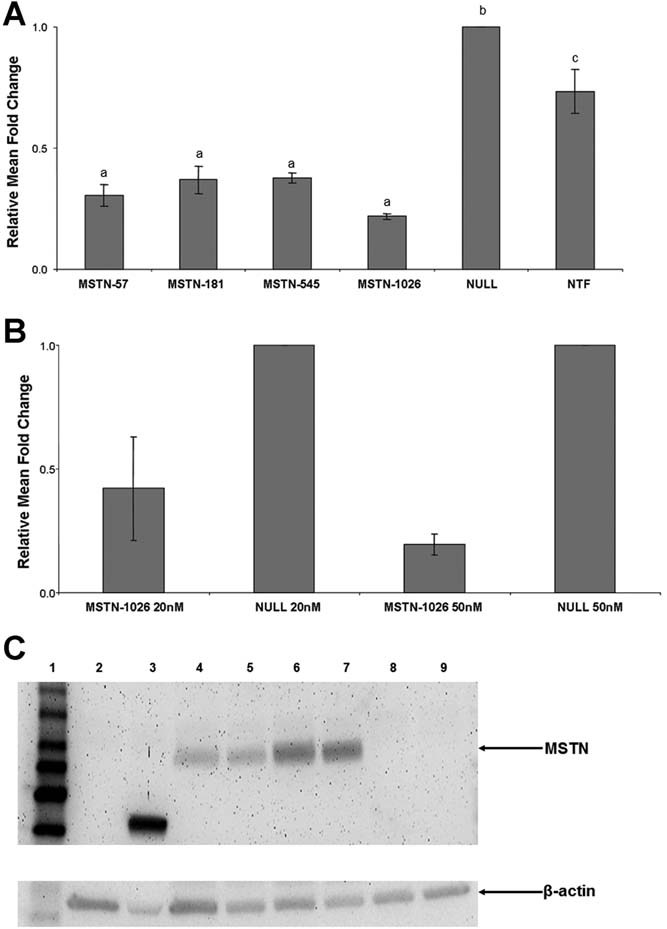
**A**: Suppression of caprine myostatin mRNA within the 293T^MSTN^ in vitro reporter line. 293T^MSTN^ cells were transfected with shRNA expressing lentiviral constructs as well as a NULL shRNA construct. Myostatin mRNA levels were analyzed by quantitative real-time PCR, and the fold-change in caprine myostatin mRNA expression was normalized to GAPDH as an internal control. Average fold-change was calculated relative to the NULL for each shRNA ± standard error of the mean (SEM), and means were compared using analysis of variance. All four shRNA expressing constructs targeting myostatin had significantly lower myostatin mRNA expression when compared to the NULL (*P* < 0.05). Means with different letters are significantly different. MSTN-57, MSTN-181, MSTN-545, and MSTN-1026, lentiviral plasmids containing shRNAs targeting myostatin; NULL, lentiviral plasmid containing a nonsense shRNA; NTF, no transfection. **B**: Suppression of bovine myostatin mRNA in isolated muscle cells. Cells were transfected with either the MSTN-1026 siRNA or the NULL siRNA (20 or 50 nM). Myostatin mRNA levels were analyzed by quantitative real-time PCR using GAPDH as an internal control. Average fold-change was calculated relative to the NULL at each concentration ± SEM, and means were compared using analysis of variance. Treatment effects within concentration were different, *P* < 0.05. **C**: Suppression of FLAG-tagged myostatin protein in HEK 293T cells. Cells were co-transfected with the FLAG-myostatin construct and either the MSTN-1026 siRNA, the Cy3 NULL siRNA, or no siRNA. Relative protein abundance was calculated using densitometry after normalization to the β-actin control, and means were compared using analysis of variance. Mean protein level for cells transfected with the MSTN-1026 siRNA was significantly lower than cells transfected with the NULL (*P* < 0.05). **Lane 1**, ladder; **Lane 2**, negative antibody control; **Lane 3**, positive antibody control; **Lanes 4** and **5**, no siRNA treatment; **Lanes 6** and **7**, NULL Cy3 treated cells; **Lanes 8** and **9**, MSTN-1026 treated cells.

Four lentiviral plasmid constructs expressing the shRNAs targeting caprine and bovine myostatin sequences (MSTN-57, MSTN-181, MSTN-545, and MSTN-1026), as well as a nonsense control shRNA construct (NULL), were transfected into a cell line designed to stably express caprine myostatin (293T^MSTN^). All four shRNAs targeting the myostatin transcript showed effective suppression of mRNA levels when compared to the NULL construct (Fig. 1A). One-way ANOVA revealed expression of myostatin mRNA in cells transfected with each shRNA targeting myostatin was significantly lower than the NULL, but not each other (*P* < 0.05).

Given the results of in vitro screening for effective shRNAs using the 293T^MSTN^ cell line, the MSTN-1026 shRNA was chosen for further testing. Due to difficulty in transducing primary muscle cells, an siRNA corresponding to the MSTN-1026 shRNA was used for evaluation. To confirm that the MSTN-1026 sequence was effective at reducing endogenous bovine myostatin, a MSTN-1026 siRNA or a NULL, Cy3-conjugated siRNA was transfected into isolated bovine muscle cells using a calcium phosphate method. This resulted in a transfection efficiency of >90% as assessed by Cy3 fluorescence. Analysis of the transfected muscle cells by quantitative PCR revealed a significant decrease in myostatin mRNA expression in cells transfected with the MSTN-1026 siRNA at both 20 and 50 nM concentrations (Fig. 1B).

### Evaluation of Myostatin Protein Suppression In Vitro

The ultimate goal with RNAi-mediated gene suppression is reduction in protein levels. In order to evaluate the effectiveness of the MSTN-1026 sequence at reducing myostatin protein expression, a FLAG-tagged myostatin construct was co-transfected with either the MSTN-1026 siRNA or the NULL, Cy3-conjugated siRNA into HEK 293T cells. A control transfection of the FLAG-myostatin construct was also performed without any siRNA to confirm expression and proper detection of the FLAG-labeled protein. Co-transfection of the MSTN-1026 siRNA with the FLAG-myostatin construct revealed a significant reduction (86%, *P* < 0.05) in myostatin protein expression when compared to a co-transfection with the NULL, Cy3-siRNA after normalization to the internal control, β-actin (Fig. 1C).

### Production of Transgenic Embryos and Offspring by Somatic Cell Nuclear Transfer

To produce transgenic offspring, somatic cell nuclear transfer (SCNT) was performed using a transgenic fetal cell line encoding the MSTN-1026 shRNA construct. A transgenic cell line modified to express the NULL shRNA was also used for cloning to produce transgenic control offspring. The lentiviral construct used for embryo production expresses GFP and the shRNA as one transcript. Therefore, evaluation of GFP expression can be used as an indicator of shRNA production in vivo. A total of 22 blastocysts were produced for each transgenic cell line (total 44). Of these, all but 1 blastocyst derived from the control (NULL) treatment were transferred into 14 recipient cows.

**Figure 2:**
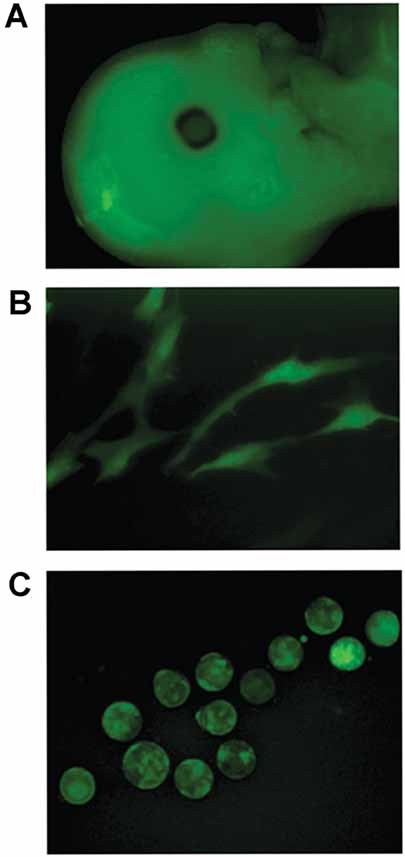
Expression of GFP in embryos, fetuses, and offspring expressing an shRNA targeting myostatin. **A**: Fetus collected at day 66 of gestation, (**B**) cells cultured from the transgenic calf produced using SCNT, and (**C**) blastocysts expressing GFP.

Ultrasounds were performed on day 42 of gestation, and 7 animals were confirmed to be pregnant (5 MSTN-1026 and 2 NULL controls, Table 1). Three fetuses were collected from a failing pregnancy at 66 days of gestation. Only one pregnancy carried to term, resulting in a single calf that died shortly after Caesarean delivery. PCR analysis of genomic DNA isolated from the fetuses and calf confirmed they were all transgenic. GFP fluorescence revealed a high level of transgene expression (Fig. 2A,B).

**Table 1:** Production and Transfer of Transgenic Embryos Using Either Somatic Cell Nuclear Transfer or Perivitelline Microinjection of In Vitro Produced Zygotes

Method of production.	No. zygotes injected	No. blastocysts	No. GFP positive	No. transferred	No. recipients	No. pregnant day 42	Day 42 pregnancy rate (%)	Fetuses collected (transgenic)	Offspring produced (transgenic)	No. expressing transgene
SCNT	n/a	44	37	43	14	7	50	3 (3)	1 (1)	3
MSTN-1026	n/a	22	20	22	7	5	71	3 (3)	1 (1)	3
NULL	n/a	22	17	21	7	2	29	0	0	0
Microinjection	1,239	195	153	85	26	13	50	2 (0)	9 (8)	4
MSTN-1026	596	88	64	40	14	7	50	2 (0)	5 (5)	3
NULL	643	107	89	45	12	6	50	0	4 (3)	1

### Production of Transgenic Embryos and Offspring by Recombinant Lentivirus Injection

**Figure 3:**
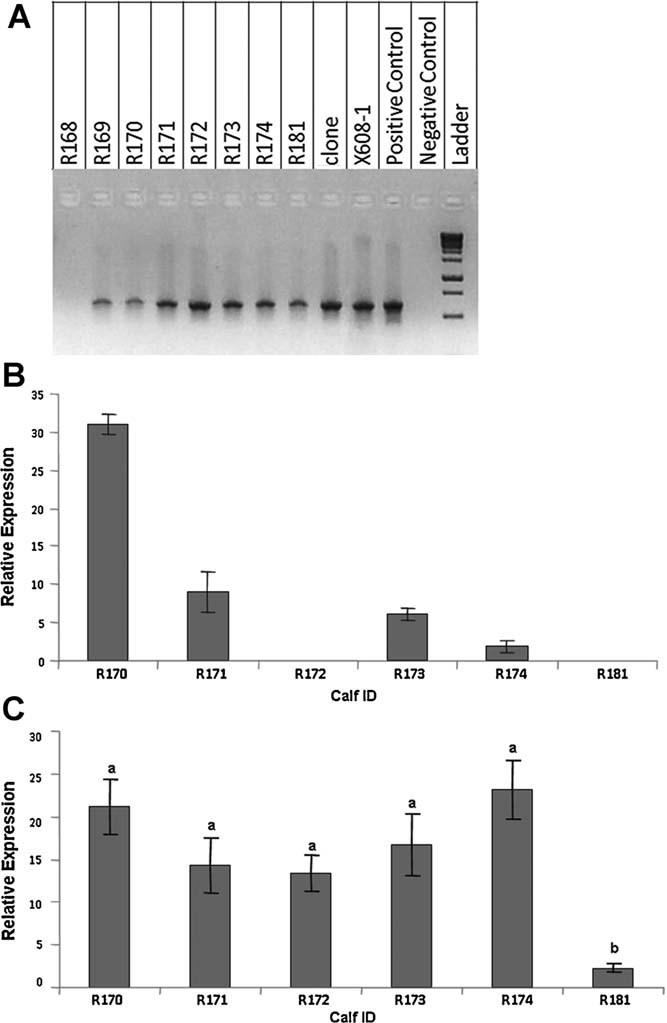
**A**: PCR analysis to detect the transgene in offspring produced using either SCNT or lentiviral microinjection. PCR was performed on genomic DNA isolated from skin with primers designed to amplify a region in the 5′ long terminal repeat (LTR) region of the transgene. R168-R174, R181, calves produced using lentiviral microinjection; clone, calf produced using SCNT; X608-1, dead calf delivered via Caesarean section and produced using lentiviral microinjection; positive control, plasmid used for lentiviral production; negative control, no DNA. Quantitative real-time PCR analysis of relative expression of GFP (**B**) and myostatin (**C**) in the Sacrocaudalis dorsalis lateralis muscle from each surviving male transgenic calf. Relative expression of the target gene was analyzed, compared to GAPDH as an internal control, and presented as the mean ± SEM, and means for myostatin were compared using analysis of variance. Means with different letters are significantly different (*P* < 0.05). R170, calf produced with the NULL shRNA; R171–174 and R181, calves produced with the MSTN-1026 shRNA.

Given the inefficiency of the SCNT procedure, we opted to derive transgenic embryos using sub-zonal microinjection of recombinant lentivirus directly into zygotes. A total of 195 blastocysts were produced in vitro following injection of high-titer, recombinant lentivirus in the perivitelline space; 153 of these were GFP positive (Table 1). Forty (40) blastocysts derived following injection of lentivirus carrying shRNA MSTN-1026 and expressing this transgene, as evidenced by GFP fluorescence (Fig. 2C), and 45 blastocysts derived following injection of the control (NULL) lentivirus were transferred into 26 recipient cows, resulting in 13 pregnancies and 9 offspring that developed to term (Table 1). When genomic DNA was analyzed from these calves, 8/9 calves were confirmed to be transgenic, 5 encoding MSTN-1026 (all males), and 3 encoding the NULL shRNA (1 male, 2 females, Fig. 3A). Between 14 and 20 months of age, muscle biopsies were collected from the Sacrocaudalis dorsalis lateralis in the five transgenic male calves encoding MSTN-1026 and one transgenic male calf encoding the NULL shRNA. Methods employed were those described by Martin et al. (1999). Analysis of mRNA isolated from muscle samples was performed using quantitative PCR to determine gene expression levels for myostatin and the transgene encoding MSTN-1026 shRNA or NULL shRNA. Expression of myostatin transcript was detected in all samples. Three calves expressed the transgene encoding shRNA designed to silence myostatin while the control calf expressed the transgene encoding the NULL shRNA (Fig. 3B,C). There was no correlation between transgene expression and the level of myostatin mRNA.

## DISCUSSION

The pronounced effects of null mutations in the myostatin gene have been well documented across species, and include hypertrophy and hyperplasia of muscle fibers (McPherron and Lee, 1997; Berry et al., 2002; Schuelke et al., 2004; Clop et al., 2006; Mosher et al., 2007; Shelton and Engvall, 2007; Boman et al., 2009). Cattle that possess a null mutation in the myostatin gene, such as Belgian Blue and Piedmontese, have an increase in calving difficulty and a high degree of offspring mortality (Bellinge et al., 2005). The ability to control the degree of suppression of myostatin in livestock may alleviate some of the negative attributes of null mutations, while at the same time increasing muscle mass and therefore meat production.

The siRNA sequences tested here were successful in suppressing myostatin expression, both when transfected in vitro as an siRNA or when expressed from a plasmid as an shRNA. A similar approach has been previously reported in zebrafish and mice, where an siRNA targeting the myostatin gene was then incorporated as an shRNA into a plasmid for in vivo transgene delivery (Magee et al., 2006; Lee et al., 2009). Therefore, we believe this approach should be effective when transitioning from in vitro testing to transgenic livestock production. In addition, the use of RNAi can add an additional level of control, as the degree of gene suppression could be manipulated through the choice of the shRNA that is introduced.

Currently, the most common method for producing transgenic livestock involves genetic modification of cell lines followed by the utilization of these for SCNT (cloning). Although effective, animal cloning remains an inefficient process, as highlighted by our experiences with this project, which resulted in a single transgenic calf that died at birth. In addition, issues involving intellectual property rights can limit the utilization of cloning for transgenic animal production. Clearly there is a great need for alternative methods for producing transgenic livestock that are effective, efficient, and economical. Difficulties with cloning prompted our use of lentiviral microinjection for transgenic embryo production. Previous work by Hofmann et al. (2003) reported an efficiency of 45% GFP-positive blastocysts when bovine zygotes were microinjected 18 hr post-fertilization, and an even a higher success rate (92%) with transduction of oocytes followed by in vitro fertilization. Milazzotto et al. (2010) also reported the injection of oocytes with lentivirus carrying a gene encoding an shRNA designed to silence the expression of myostatin. After fertilization, however, only 3.07% of the hatching blastocysts were GFP-positive (Milazzotto et al., 2010). In the current study, microinjection of lentivirus into zygotes 8–10 hr post in vitro fertilization resulted in 78% of the blastocysts expressing GFP. Additionally, transfer of transgenic blastocysts produced through microinjection produced a similar pregnancy rate as SCNT at 35 days gestation (50%), but resulted in the live birth of 7 transgenic calves versus 0 from SCNT. This clearly demonstrates the increased efficiency of transgenic animal production with this method over cloning.

**Figure 4:**
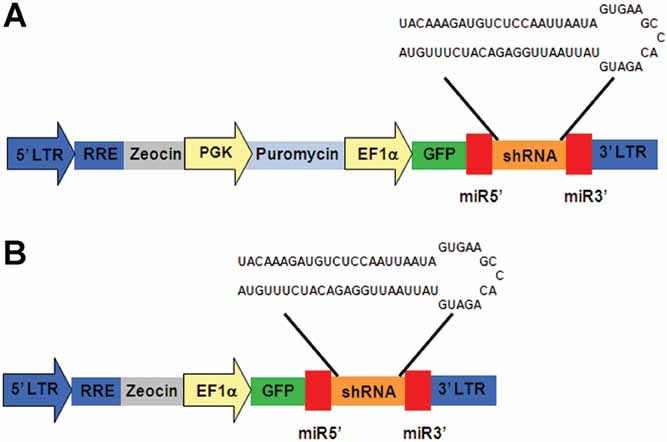
Graphic representation of the lentiviral plasmids used in this experiment. **A**: Lentiviral plasmid used to produce transgenic donor cells for somatic cell nuclear transfer. This plasmid contains a puromycin resistance gene for antibiotic selection of transgenic cells, and the elongation factor 1 alpha (EF1α) promoter driving expression of GFP and the shRNA. The shRNA sequence listed corresponds to MSTN-1026. **B**: Lentiviral plasmid used to produce recombinant lentivirus for microinjection into zygotes. This plasmid is the same as in (A), but with the antibiotic resistance removed. LTR, long terminal repeat; RRE, rev response element; PGK, phosphoglycerate kinase promoter; GFP, green fluorescent protein.

The constructs used in these experiments were designed such that transcripts encoding the GFP reporter and the shRNA were produced as a single messenger RNA (Fig. 4). Accordingly, GFP transcription and fluorescence levels are both strong indicators of shRNA production (Golding and Mann, 2011). In these studies, analysis of transgene expression in embryos, fetuses, and live calves involved both observations for GFP fluorescence and/or quantitative real-time PCR analysis of GFP mRNA. Blastocysts were confirmed to be expressing the transgene prior to embryo transfer. Collected fetuses also expressed the transgene, as did the cloned calf and at least four of the live calves produced by injection of lentivirus into zygotes (3 derived using MSTN-1026 shRNA and 1 NULL control).

Considerable variation in transgene expression levels was observed between different calves, and expression of the transgene was undetectable in at least two cases. The significance of this, as related to our overall goals for this project, is tempered by the likelihood that transgene expression varies between animals relative to their developmental clock. Previous studies by our group and others have suggested the EF1α promoter used in these constructs is both highly expressed and epigenetically stable in embryonic and extraembryonic tissues (Golding and Mann, 2011). Furthermore, transgenic mice produced through sub-zonal injection of lentiviral particles exhibited strong transgene expression in a variety of tissues over six generations (Kvell et al., 2010). The strength and stability of this promoter in cattle, however, has not been examined until now. Myostatin mRNA is normally produced by the developing somites and is detected by RT-PCR as early as day 29 in cattle (Kambadur et al., 1997). Primary myotube development is established by day 39 of gestation in cattle, and secondary myofiber differentiation is established by day 90 (Oldham et al., 2001). Therefore, it is expected that expression of transgenes designed to silence the expression of myostatin during this particular time of gestation would result in increased muscle development in the resulting fetuses and calves. We confirmed that embryos were expressing the transgene at the time of embryo transfer, but we do not know the status of gene expression during fetal development of the transgenic calves. The possibility exists that those calves that did not exhibit gene expression after birth did express the transgene during fetal development.

**Figure 5:**
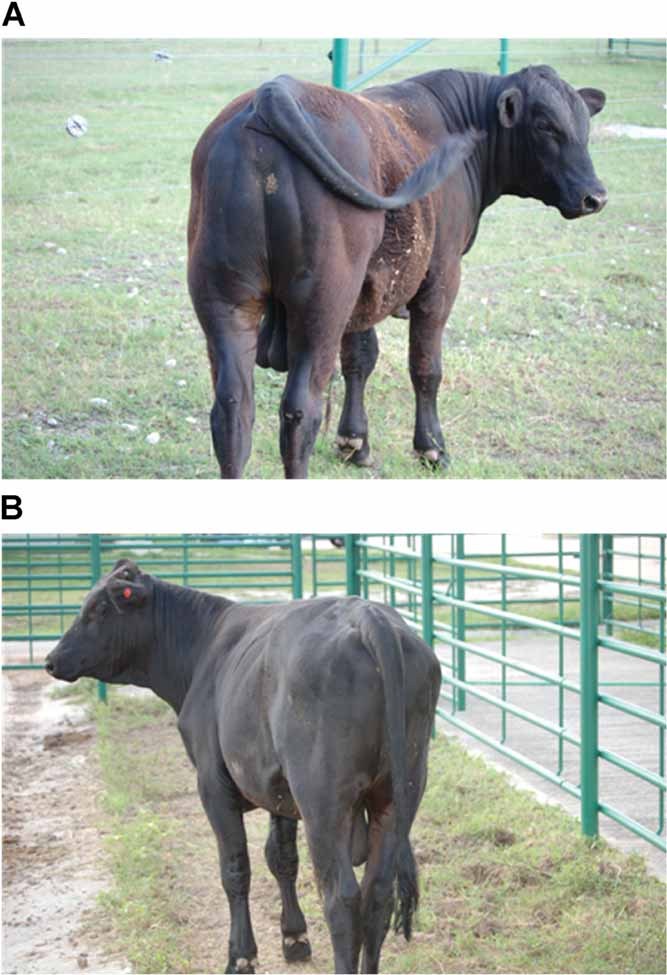
Genetically modified calves with a transgene encoding (**A**) shRNA MSTN-1026 targeting the myostatin gene or (**B**) a NULL shRNA at 14 months of age. The same Brangus sire was used for in vitro fertilization in the production of these two calves; the genetic background of the dams, however, is unknown.

Figure 5 depicts two transgenic bulls, one with the gene encoding shRNA MSTN-1026 (R181) and the second encoding the NULL shRNA (R170, control). Both of these bulls are 14 months old and both were derived from the same Brangus sire. The transgenic bull encoding MSTN-1026 obviously appears to exhibit more muscle mass when compared to the transgenic NULL. This bull also exhibits the lowest level of myostatin mRNA when compared to other bulls; unfortunately, we were unable to detect transgene expression in muscle derived from this animal (Fig. 3B). The control bull (R170) expressed the transgene encoding a nonsense shRNA. The increased muscle development exhibited in bull R181 might represent an example where the shRNA targeting myostatin was expressed during fetal development and not in the adult. At this point, however, there is no way to resolve this uncertainty. To date we have not produced enough transgenic calves to demonstrate any association between transgene expression and phenotype. Analysis of muscle samples derived from the transgenic calves does not support a correlation between expression of MSTN-1026 and myostatin, even though in vitro studies clearly demonstrate this shRNA sequence to be highly effective at silencing myostatin gene and protein expression.

All the calves produced in these studies were derived from ova collected from a slaughterhouse, and we have no information on their dams beyond the general genetic background of cows normally processed at this facility being of dairy breed descent (Holstein). Differences in muscle development exhibited between these two animals could be explained by their genetic background (parents) or by environmental/epigenetic factors. Additional experiments will have to be conducted and more animals produced to thoroughly test this hypothesis.

In conclusion, here we report the production of transgenic calves expressing an shRNA that effectively reduces myostatin mRNA and protein expression in vitro. Both SCNT and lentiviral microinjection resulted in a high percentage of embryos expressing the transgene, although lentiviral microinjection proved more efficient in the production of live offspring. Work is currently underway to use these cattle for breeding studies to determine the inheritance patterns of the transgene, in addition to gene expression and the extent of myostatin suppression and muscle characteristics in these animals. Production of transgenic livestock expressing an shRNA targeting the myostatin gene should result in animals with enhanced muscle development. This could increase the efficiency of food production agriculture, and analysis of such animals could also contribute to the development of human therapeutics for the treatment of muscle wasting disorders.

## MATERIALS AND METHODS

### Design and Production of Gene Constructs Encoding shRNAs

Given our goal to perform similar experiments in both goats and cattle, bovine and caprine myostatin coding sequences were aligned using the ClustalW alignment program (http://www.clustalw.genome.ad.jp) and regions of sequence homology were identified. These regions were loaded into an siRNA design program at http://www.biopredsi.org. Resulting sequences were then checked for nonspecific targets using BLAST. To enhance proper processing of short hairpin RNA (shRNA) sequences by Drosha, the myostatin siRNA sequences were modified for cloning by adding microRNA30 (mir30) sequences using methods described by (Paddison et al., 2004) (http://hannonlab.cshl.edu/GH_shRNA.html). Myostatin shRNA mir30 amplicons were cloned into the 3′ UTR region of the enhanced green fluorescent protein (eGFP) within the PEG vector, as previously described (Golding et al., 2010). The PEG lentiviral plasmid is composed of the EF1 alpha promoter driving expression of a gene encoding an shRNA designed to silence the expression of myostatin in addition to GFP (Fig. 4). Four different lentiviral plasmid constructs expressing shRNAs targeting caprine and bovine myostatin sequences (MSTN-57, MSTN-181, MSTN-545, and MSTN-1026), as well as a nonsense control shRNA construct (NULL), were produced and tested for their ability to silence the expression of myostatin.

### Production of Recombinant Lentivirus

Recombinant lentivirus was produced as described by Miyoshi et al. (1998). A representation of the recombinant lentiviral plasmids utilized for cells growing in vitro (A) and injection of zygotes to produce transgenic calves (B) is provided in Figure 4. The vector expresses an shRNA in addition to GFP. Briefly, lentiviral plasmid (PEG shRNA) was co-transfected into HEK 293T cells along with pCMV R8.91 (packaging plasmid) and pMDG (vesicular stomatitis virus glycoprotein plasmid) via calcium phosphate transfection, as described previously (Golding et al., 2010). Media was changed 24 hr post-transfection and supernatant collected 48 hr later. Supernatants were centrifuged (2,000*g* for 20 min) to remove cellular material and subsequently filtered through a 0.45 µm filter. Supernatant containing the recombinant lentiviral particles was either stored at 4°C until use or frozen in aliquots at −80°C. Recombinant lentivirus intended for embryo microinjection was concentrated using ultracentrifugation prior to use. Viral supernatant was ultracentrifuged for 1.5 hr at 50,000*g* at 4°C. Supernatant was removed, and viral pellets were resuspended in 20–30 µl sterile phosphate-buffered saline (PBS) with 8 µg/ml polybrene overnight at 4°C before being frozen at −80°C in 5 µl aliquots.

The concentration of infective recombinant virus was determined using GFP fluorescence. HEK 293T cells were plated at a density of 25,000 cells/well in a 48-well plate, and transduced approximately 8 hr later with serial dilutions of concentrated recombinant lentivirus. Due to limited volumes of concentrated virus, serial dilutions of 0.1, 0.01, 0.001, and 0.0001 were used. All transductions were done with 8 µg/ml polybrene. Media was refreshed 18 hr later, and cells were maintained in culture for 3 days before evaluation. Recombinant lentivirus titer was calculated as follows: [(No. of cells plated) × (% GFP positive cells)]/(ml viral media) × dilution factor.

### Evaluation of Myostatin mRNA Suppression In Vitro

The caprine myostatin coding sequence (AY436347) was amplified from goat muscle cDNA and cloned into the pcDNA3.1 plasmid (Invitrogen, Carlsbad, CA). The coding sequence was PCR amplified using primers designed to add *Eco*RI and *Bam*H1 restriction sites on each end for cloning into the pEIT lentiviral plasmid (Welm et al., 2008). This plasmid contains an EF1 alpha promoter followed by a multiple cloning site, as well as an internal ribosomal entry site (IRES) and the tomato red fluorescent protein. Recombinant lentivirus was produced as described, and used to transduce HEK 293T cells. Transgenic, myostatin expressing 293T cells (293T ^MSTN^) were confirmed by detection of the red fluorescent protein using fluorescence microscopy. 293T ^MSTN^ cells were plated into 6-well plates and transfected with recombinant lentiviral plasmids containing either an shRNA targeting MSTN or a nonsense control shRNA (NULL). Cells were maintained in culture for 4 days before being harvested for RNA isolation using the RNeasy kit, as per manufacturer's protocol (Qiagen, Germantown, MD).

Quantitative real time PCR was utilized to compare different shRNAs and null controls for their ability to suppress myostatin mRNA. Briefly, RNA was quantified using a spectrophotometer, and 0.2–0.5 µg RNA was added to a 20 µl reaction to synthesize cDNA using either the qScript cDNA synthesis kit (Quanta Biosciences, Gaithersburg, MD) or Superscript III cDNA Synthesis kit (Invitrogen) according to their respective manufacturers' protocols. Quantitative real-time PCR (qPCR) reactions were set up in triplicate using either Power SYBR Green MasterMix (Applied Biosystems, Foster City, CA) or Perfecta SYBR Green FastMix (Quanta Biosciences) and 2 µM primer mix. Cycling conditions were as follows: 95°C for 20 sec, 40 cycles of 95°C for 3 sec, and 60°C for 30 sec. A melt curve analysis followed, with temperature increments of 0.3°C from 60°C to 95°C. To determine primer efficiency, a relative standard curve was analyzed using a dilution series of the appropriate cDNA. Quantitative real time PCR data was analyzed using the ΔΔCt method with comparison to GAPDH as an internal control. The mean fold-change was calculated for each replicate, and then divided by the mean fold-change for the control to obtain a relative fold-change. The relative mean fold-change for each treatment group was compared to the control using one-way ANOVA with a Tukey test for comparison of means when applicable.

To ensure that the designed siRNA sequences would also be equally effective in cattle in vivo, an additional evaluation was performed using primary bovine muscle cells. Muscle cells were isolated from adult tissue explants as previously described with the following modifications (Cassar-Malek et al., 1999). Skeletal muscle tissue was plated in collagen-coated 25 mm^2^ flasks, 3–4 pieces per flask, and cultured in DMEM/F12 with 20% FBS, 5 ng/ml basic fibroblast growth factor, and gentamicin (Invitrogen). Media was refreshed one day later and flasks were maintained at 37°C with 5% CO_2_. At the first passage, cells were preplated for 20 min at 37°C to enrich for myoblast cells. To induce fusion into myotubes, cells were first plated in fibronectin-coated wells and then switched to a low serum media containing 2% horse serum.

Due to difficulties with transfecting large plasmids into primary muscle cells and inefficient transduction, siRNA sequences for the MSTN-1026 and NULL were ordered from Ambion (Austin, TX) for transfection. The NULL siRNA was designed with a Cy3-label conjugated to it for evaluation of transfection efficiency. One day prior to transfection, early passage bovine muscle cells were plated at a density of 5 × 10^4^ cells on fibronectin-coated 12-well plates. On the day of transfection, either 20 or 50 nM of siRNA (MSTN-1026 or Cy3 conjugated negative control) was combined with 3.5 µl 2 M CaCl_2_ in a 30 µl volume, and then an equal volume of 2× HBS was added. Cells were maintained in culture for 4 days, after which RNA was isolated and qPCR utilized to compare siRNAs and null controls for their ability to suppress myostatin mRNA using methods similar to those described above.

### Evaluation of Myostatin Protein Suppression In Vitro

Effective antibodies for detection of bovine or caprine myostatin were unavailable; therefore to confirm that our shRNAs were capable of suppressing myostatin protein, the caprine myostatin coding sequence (AY436347) was cloned into the pcDNA3.1 plasmid (Invitrogen). A FLAG tag sequence was added onto the coding sequence using PCR to allow detection by Western blot using an anti-FLAG antibody. In order to measure protein reduction in experiments utilizing siRNAs, co-transfections were performed in HEK 293T cells. For each replicate, 3 µg FLAG-tagged myostatin plasmid and 50 nM siRNA (MSTN-1026 or Cy3-conjugated negative control) were co-transfected using calcium phosphate. In addition, a control replicate was transfected containing only the FLAG-tagged myostatin. Cells were cultured for 3 days prior to being lysed for protein extraction. Lysate was collected and centrifuged at 4°C for 30 min at 15,000*g*, and the supernatant was quantified using a Bradford assay. Protein, 10 µg for each treatment group, was loaded onto a precast 4–20% polyacrylamide gel (Biorad). After electrophoresis, protein was transferred onto a PVDF membrane and blocked using 5% milk in Tris-buffered saline with 0.1% Tween-20 (TBST). Primary anti-FLAG antibody (Rockland Immunochemicals for Research, Gilbertsville, PA) was diluted 1:3,000 in 5% milk-TBST and rocked overnight at 4°C. After rinsing with TBST 3 times, horseradish-peroxidase-conjugated anti-rabbit secondary antibody (Abcam, Cambridge, MA) was diluted 1:10,000 in 5% milk TBST and added. The blot was rocked for 1 hr at room temperature. After rinsing with TBST 3 times, chemiluminescent substrate (Thermo Fisher Scientific, Waltham, MA) was added for 5 min and the blot was imaged. After imaging, the blot was rinsed with double distilled water and then stripped for relabeling. The same procedure as above was performed using anti-β actin primary antibody (Abcam, Cambridge, MA). Spot densitometry was performed to determine relative protein levels, and a mean ratio was calculated for each treatment group. Relative protein suppression was calculated by comparison to the mean ratio for cells treated with the NULL Cy3-conjugated siRNA. One-way ANOVA was performed to test for significant differences among treatment groups.

### Production of Transgenic Embryos Using Somatic Cell Nuclear Transfer (SCNT)

Recombinant lentivirus encoding GFP and the most effective shRNA, as determined by in vitro experiments, was produced as described above. Lentivirus encoding a nonsense control shRNA was also produced. Medium containing recombinant lentiviral particles was used to transduce a bovine fetal fibroblast cell line. Cells were plated at 70–80% confluence in 6-well plates, and lentivirus-containing medium added to the cells for 16–20 hr. Once fluorescence was visualized, cells were placed under antibiotic selection (1 µg/ml puromycin). Approximately 100% of cells were GFP positive after 3–4 days. These cells were further cultured to expand the line and cryopreserved for future use. Transgenic cell lines were sent to ViaGen, Inc. in Austin, Texas to produce cloned blastocysts by somatic cell nuclear transfer.

### Production of Transgenic Embryos by Microinjection of Zygotes With Recombinant Lentivirus

Bovine embryos were produced using in vitro fertilization, as previously described with modifications (Parrish et al., 1986). Fertilized zygotes were removed from fertilization media 7.5 hr post-insemination and vortexed for 2 min in TL Hepes with 5 µg/ml hyaluronidase to remove cumulus cells. Presumptive zygotes were placed in a 100 mM sucrose solution in M199 Hanks Hepes media (Invitrogen) during micromanipulation.

Recombinant lentivirus was produced as described above (Miyoshi et al., 1998; Golding et al., 2010), and viral supernatant was ultracentrifuged prior to microinjection to increase titer. Only concentrated virus with a titer at or above 1 × 10^9^ IU/ml was used for microinjection of embryos. Microinjection of recombinant lentiviral particles beneath the zona pellucida was performed between 8 and 10 hr post-fertilization. Perivitelline injections were performed with a micropipette using a steady flow into the perivitelline space for 20–30 sec per embryo. Embryos were then cultured in groups of up to 50 embryos in 500 µl Evolve bovine formulated culture media (Zenith Biotech, Guilford, CT) plus 4 mg/ml Probumin (Millipore, Jaffrey, New Hampshire) and 100 µg/ml gentamicin. At day 7 of culture, blastocysts were evaluated for GFP fluorescence.

### Embryo Transfer and Evaluation of Transgenic Calves

Grade-1 and -2 embryos expressing GFP were either transferred into synchronized recipient cattle on day 7 of culture or cryopreserved prior to transfer. A total of 2–4 embryos were transferred per recipient, and pregnancy diagnosis was conducted using ultrasound on day 42 of gestation.

Shortly after birth, each calf was subjected to an ear biopsy. Genomic DNA was isolated from each using the DNeasy kit (Qiagen). PCR was performed on 20 ng of genomic DNA using primers designed to amplify a region of the integrated transgene in the 5′ long terminal repeat (LTR) region.

In order to analyze transgene expression and suppression of myostatin in transgenic calves, biopsies were taken from the Sacrocaudalis dorsalis lateralis muscle, as previously described (Martin et al., 1999). Quantitative real-time PCR was employed to analyze GFP (as a measure of transgene transcription) as well as myostatin mRNA expression after normalization to GAPDH as an internal control. The correlation between GFP and myostatin expression was evaluated by calculating a Pearson correlation coefficient, which was tested against the null hypothesis that no correlation exists using a *P*-value of 0.05.

## Abbreviations

MSTN: myostatin
RNAi: RNA interference
SCNT: somatic cell nuclear transfer
siRNA: short interfering RNA
shRNA: short hairpin RNA
